# The Associations between RNA Splicing Complex Gene *SF3A1* Polymorphisms and Colorectal Cancer Risk in a Chinese Population

**DOI:** 10.1371/journal.pone.0130377

**Published:** 2015-06-16

**Authors:** Xiaohua Chen, Hua Du, Binjian Liu, Li Zou, Wei Chen, Yang Yang, Ying Zhu, Yajie Gong, Jianbo Tian, Feng Li, Shan Zhong

**Affiliations:** 1 Department of Medical Genetics, School of Basic Medical Science, Wuhan University, Wuhan, 430071, China; 2 Department of Laboratory Medicine, No 161 Hospital of PLA, Wuhan, 430010, China; 3 Department of Epidemiology and Biostatistics, School of Public Health, Tongji Medical College, Huazhong University of Science and Technology, Wuhan, China; 4 Hubei Provincial Key Laboratory of Developmentally Originated Disease, Wuhan, 430071, China; Beijing University of Chemical Technology, CHINA

## Abstract

**Background:**

Aberrant alternative splicing included alterations in components of the mRNA splicing machinery often occurred in colon cancer. However, the role of *SF3A1*, one key component of the mRNA splicing machinery, on colorectal cancer (CRC) risk was still not elucidated.

**Method and Findings:**

We performed a hospital-based case-control study containing 801 CRC patients and 817 cancer-free controls to examine the association between *SF3A1* polymorphisms and CRC risk in a Chinese population. Four candidate SNPs (rs10376, rs5753073, rs2839998 and rs2074733) were selected based on bioinformatics analysis and previous findings. The results showed no significant associations between these SNPs and CRC risk (*P* > 0.05). Besides, the stratified analysis based on the smoking and alcohol use status obtained no statistically significant results.

**Conclusion:**

Our study was the first one to investigate the association between *SF3A1* polymorphisms and CRC risk. The results suggested these four SNPs in *SF3A1* were not associated with CRC risk in a Chinese population, however, further more studies are needed to confirm our findings.

## Introduction

Colorectal cancer (CRC) remains a major health problem and is a leading cause of morbidity and mortality worldwide, representing the third most common cause of cancer-related death[1]. It was estimated to cause 142,820 new cases and 50,830 deaths of the colon and rectum cancer in the United States for both men and women in 2013[2]. Although several environmental risk factors [3,4] have been detected to be associated with risk of CRC, genetic susceptibility was found to be involved in the development of this disease. Genome-wide association studies (GWAS) have been successful applied in identifying susceptibility loci for cancer and other diseases [5,6]. In colorectal cancer, recent GWAS studies have revealed more than 20 susceptibility single nucleotide polymorphisms (SNPs) in multiple different loci in European and Asian populations [7–20]. However, most of them are located in non-coding regions and can explain less than 10% of the familial relative risk of CRC in European populations [13,14].These indicated that there may be a substantial fraction of genetic components undiscovered and the biological mechanisms are required to be explored.

RNA splicing can remove introns from pre-messenger RNAs and is essential to all eukaryotic organisms to generate considerable numbers of alternative isoforms with altered coding potential or regulatory regions in order to guarantee the functional diversity of their protein in the face of a limited number of genes[21,22]. However, aberrant alternative splicing resulted from mutations within splicing elements in cancer genes or transcripts from non-mutated genes occurred in many cancers [23]. For example, a few studies have investigated the aberrant alternative splicing in colon cancer and detected many colon cancer specific alternative splicing events affecting several proteins or pathways[24–28]. These colon cancer-related splicing events often involved alterations in components of the mRNA splicing machinery, which was exemplified by the recent finding that amplification or overexpression of *PRPF6* could be a driver of colon tumorigenesis[29].

RNA splicing is a well-ordered process that recruits, rearranges and disengages of a set of small nuclear ribonucleoprotein (snRNP) complexes, as well as many other protein components onto the pre-mRNAs. During splicing, SF3A1, together with the U2 snRNP and other proteins, are recruited to the 3’ splicing site to generate the splicing complex A after the recognition of the 3’ splicing site[30].Therefore, *SF3A1* is critical for spliceosome assembly and normal splicing events. *SF3A1* is located in 22q12.2, where has been reported to be associated with susceptibility of lung cancer [31], breast cancer [32] and inflammatory bowel disease [33] by genome-wide association studies. Several studies have reported the associations between mutations of *SF3A1* and other diseases. Yoshida et al[30] have recently discovered lower mutational rates for *SF3A1* in the majority of the patients with myelodysplastic syndromes (MDS). Additionally, curated information from the Catalogue of Somatic Mutations in Cancer (COSMIC) database revealed that mutations in the coding-region of *SF3A1* were associated with several cancers, including esophageal adenocarcinoma, myxoid liposarcomas, synovial sarcomas, osteosarcomas, endometrial tumors, lung cancer, breast cancer, ovarian carcinoma, gastric cancer and glioblastoma. Collectively, these findings suggested the link between *SF3A1* and cancer risk, and emphasized a need of additional researches for the association of *SF3A1* polymorphisms and CRC risk.

Considering the common occurrence of aberrant alternative splicing in CRC and the role of *SF3A1* in alternative splicing, we hypothesized the polymorphisms of *SF3A1* might also contribute to the susceptibility of CRC. In the present study, we carried out a hospital-based case-control study in a Chinese population to investigate the association between polymorphisms of *SF3A1* and CRC risk.

## Materials and Methods

### Ethics Statement

At the recruitment, written informed consent was obtained from each subject. The personal information about sex, birth year, smoking and drinking habits of all participants were also collected by interviews. Meanwhile, 5ml peripheral blood sample from each subject was collected and stored in the -80°C refrigerator before DNA extraction. This study was approved by ethics committee of Tongji Hospital of Huazhong University of Science and Technology.

### Study participants

A total of 801 CRC cases and 817 cancer-free controls were investigated in this study, all of whom were unrelated ethnic Han Chinese living in Wuhan region. Patients who had been histopathologically confirmed with primary colorectal cancer were enrolled from Tongji Hospital between 2009 and 2013, and had not received radiotherapy or chemotherapy before blood samples collection, part of which were described in our previous studies[34–36]. Cancer-free controls were selected from physical examination participants in the same hospital and during the same period without any history of cancer or seriously chronic disease. The control subjects were frequency matched to CRC patients by age (±5 years) and gender.

### Identification of candidate SNPs

The candidate SNPs were identified based on bioinformatics analysis and related findings. The screening procedure was described as follows. First, we input the gene “*SF3A1*” into a web-based bioinformatics tool “SNPinfo—SNP Function Prediction” (http://snpinfo.niehs.nih.gov/snpinfo/snpfunc.htm) with allele frequency restricted to “CHB” and “Asian” populations. The tool integrates GWAS and candidate gene information to predict functional characteristics of both non-coding and coding SNPs, such as transcription-factor-binding site (TFBS), microRNA-binding site, splice site, regulatory potential score, Polyphen and so on. As a result, 32 SNPs with MAF of CHB or Asian > 5% were retrieved, among which only rs10376 and rs5753073 were predicted to locate in the binding sites of microRNA with the miRanda scores for two alleles differed by ≥ 16. Then, another bioinformatics tool “miRNA SNP v2.0” (http://bioinfo.life.hust.edu.cn/miRNASNP2/) was adopted to predict the function of rs2839998 because of its ambiguous result by “SNPinfo—SNP Function Prediction”. As expected, the SNP was also predicted to be a microRNA-binding site. Besides, the SNP of rs2074733 was revealed to be associated with increased risk of pancreatic cancer in our previous study. Therefore, four SNPs (rs10376, rs5753073, rs2839998 and rs2074733) were finally selected in our study.

### DNA isolation and genotyping

5ml peripheral blood sample from each case and control subject was used to isolate genomic DNA by using RelaxGene Blood System DP319-02 (Tiangen, Beijing, China) according to the manufacturer’s instructions. The 7900HT Fast Real-Time PCR System (Applied Biosystems, Foster city, CA) was applied to determine the genotypes of rs2074733, rs10376, rs2839998 and rs5753073 by using the TaqMan SNP Genotyping Assay. (Applied Biosystems, Foster City, CA). Amplification was done under the following conditions: 95°Cfor 10 min followed by 45 cycle of 94°C for 30 s and 62°C for 1 min. Data were analyzed using Allelic Discrimination Program (Applied Biosystems). In addition, we randomly selected 5% samples and genotyped twice as quality control with a concordance rate of 100%.

### Statistical analysis

To evaluate the differences in distribution of sex, age, smoking, drinking status and genotypes between case and control groups, Pearson χ^2^ test and *t* test were employed where appropriate. Hardy–Weinberg equilibrium for genotypes was tested in controls by a goodness-of-fit χ^2^-test. The associations between rs2074733, rs10376, rs2839998 and rs5753073 and CRC risk were evaluated by the OR and its 95% confidence interval (95% CI) using unconditional logistic regression analysis adjusted by age, sex, smoking habit and alcohol use. SPSS 12.0 software was used to perform the two sided statistical analyses and *P* < 0.05 was considered statistically significant.

## Results

### Characteristics of the study population

In the present study, we have recruited 801 CRC patients and 817 cancer-free healthy individuals, whose characteristics are shown in **Table 1**. The mean age of patients and controls were 58.18 years (±11.68) and 57.51 years (±11.44), respectively. Among cases 58.4% were males compared with 58.0% among controls. Additionally, 35.3% cases were smokers and 31.6% cases with drinking habits. There were no significant differences in the distribution of age (*P* = 0.244), sex (*P* = 0.867), smoking habit (*P* = 0.122) and alcohol use (*P* = 0.453) between case and control groups.

**Table 1:** Distribution of characteristics among cases and controls.

Variables	Case (N = 801) No. (%)	Control (N = 817) No. (%)	*P*
Age (years)	58.18±11.68	57.51±11.44	0.244 ^a^
Sex			0.867 ^b^
Male	468(58.4)	474(58.0)	
Female	333(41.6)	343(42.0)	
Smoking			
Yes	283 (35.3)	259 (31.7)	0.122 ^b^
No	518 (64.7)	558 (68.3)	
alcohol use			
Yes	253 (31.6)	244 (29.9)	0.453^b^
No	548 (68.4)	573 (70.1)	

a: *P* value was calculated by *t* test

b: *P* value was calculated by *χ* ^2^ test

### Association analysis

Genotype distributions of the *SF3A1* polymorphisms in the CRC patients and control individuals are shown in **Table 2**. The genotypes of the rs5753073, rs2839998, rs10376 and rs2074733 in controls conformed to Hardy-Weinberg equilibrium (HWE) with *P* values of 0.802, 0.734, 0.668 and 0.208, respectively. The logistic regression analysis showed no significant associations between the heterozygote and homozygote variations of all these 4 SNPs and CRC risk after adjusted by age, sex, smoking habit and alcohol use. For example, individuals with rs5753073 GG or GA genotype showed similar CRC risk compared to those with the AA genotype (OR = 0.73, 95% CI: 0.37–1.44 and OR = 0.86, 95% CI: 0.68–1.08). Similarly, there were no differences in the genotype distributions of rs2839998, rs10376 and rs2074733 between cases and controls.

We next stratified our study subjects to investigate the relationship of the *SF3A1* polymorphisms with smoking and alcohol use status. However, we still observed no significant associations between these SNPs and CRC risk in smoking and alcohol use subgroups (*P*>0.05) (**Tables 3 and 4**).

**Table 2:** Association between selected polymorphisms and CRC risk.

*SF3A1*- genotype	Case (N = 801) No. (%)	Control (N = 817) No. (%)	*P*-HWE	MAF in 1000 genomes project	MAF in Case	MAF in Control	*P* ^a^	*P* ^b^	OR ^b^(95% CI)
rs5753073			0.802	0.120	0.134	0.153			
AA	594 (75.0)	584 (71.8)					0.325		1.00
AG	183 (23.1)	209 (25.7)						0.183	0.86 (0.68–1.08)
GG	15 (1.9)	20 (2.5)						0.361	0.73 (0.37–1.44)
A/G							0.132	0.119	0.85 (0.70–1.04)
Additive^c^								0.120	0.86 (0.70–1.04)
rs2839998			0.734	0.350	0.337	0.315			
GG	340 (44.6)	368 (46.7)					0.288		1.00
GA	330 (43.3)	344 (43.7)						0.715	1.04 (0.84–1.29)
AA	92 (12.1)	76 (9.6)						0.105	1.32 (0.94–1.86)
G/A							0.180	0.166	1.11 (0.96–1.29)
Additive								0.168	1.11 (0.96–1.29)
rs10376			0.668	0.070	0.093	0.105			
CC	658 (83.0)	640 (80.2)					0.256		1.00
AC	123 (15.5)	148 (18.5)						0.124	0.81 (0.63–1.06)
AA	12 (1.5)	10 (1.3)						0.760	1.14 (0.49–2.66)
C/A							0.235	0.241	0.87 (0.69–1.10)
Additive								0.252	0.88 (0.70–1.10)
rs2074733			0.208	0.470	0.467	0.467			
TT	221 (27.9)	221 (27.4)					0.884		1.00
TC	402 (50.8)	420 (52.0)						0.669	0.95 (0.75–1.20)
CC	169 (21.3)	167 (20.7)						0.917	1.02 (0.76–1.35)
T/C							0.973	0.962	1.00 (0.87–1.15)
Additive								0.962	1.00 (0.87–1.16)

a: *P* values were calculated by *χ* ^2^ test

b: Adjusted by age, sex, smoking and alcohol use

c: Additive model

**Table 3:** Subgroup analysis according to the smoking status.

	No Smokers				Smokers			
*SF3A1*- genotype	Case (N = 518) No. (%)	Control (N = 558) No. (%)	*P* ^a^	OR (95% CI)	Case (N = 283) No. (%)	Control (N = 259) No. (%)	*P* ^a^	OR (95% CI)
rs5753073								
AA	387 (75.3)	406 (73.0)		1.00	207 (74.5)	178 (69.3)		1.00
AG	116 (22.6)	138 (24.8)	0.486	0.90 (0.68–1.20)	67 (24.1)	71 (27.6)	0.277	0.80 (0.54–1.19)
GG	11 (2.1)	12 (2.2)	0.883	0.94 (0.41–2.16)	4 (1.4)	8 (3.1)	0.141	0.40 (0.12–1.36)
rs2839998								
GG	219 (44.4)	254 (47.1)		1.00	121 (45.0)	114 (45.8)		1.00
GA	215 (43.6)	229 (42.5)	0.514	1.09 (0.84–1.42)	115 (42.8)	115 (46.2)	0.744	0.94 (0.65–1.36)
AA	59 (12.0)	56 (10.4)	0.339	1.22 (0.81–1.84)	33 (12.3)	20 (8.0)	0.169	1.55 (0.83–2.87)
rs10376								
CC	424 (82.8)	436 (79.4)		1.00	234 (83.3)	204 (81.9)		1.00
AC	82 (16.0)	106 (19.3)	0.149	0.79 (0.58–1.09)	41 (14.6)	42 (16.9)	0.560	0.87 (0.54–1.40)
AA	6 (1.2)	7 (1.3)	0.776	0.85 (0.28–2.57)	6 (2.1)	3 (1.2)	0.489	1.65 (0.40–6.74)
rs2074733								
TT	148 (28.9)	156 (28.4)		1.00	73 (26.1)	65 (25.2)		1.00
TC	250 (48.8)	281 (51.1)	0.714	0.95 (0.71–1.26)	152 (54.3)	139 (53.9)	0.779	0.94 (0.63–1.42)
CC	114 (22.3)	113 (20.5)	0.683	1.08 (0.76–1.52)	55 (19.6)	54 (20.9)	0.625	0.88 (0.53–1.47)

a: Adjusted by age, sex and alcohol use

**Table 4:** Subgroup analysis according to the drinking status.

	No Drinkers				Drinkers			
*SF3A1*- genotype	Case (N = 548) No. (%)	Control (N = 573) No. (%)	*P* ^a^	OR (95% CI)	Case (N = 253) No. (%)	Control (N = 244) No. (%)	*P* ^a^	OR (95% CI)
rs5753073								
AA	414 (76.2)	416 (73.0)		1.00	180 (72.3)	168 (69.1)		1.00
AG	118 (21.7)	141 (24.7)	0.464	0.86 (0.57–1.30)	65 (26.1)	68 (28.0)	0.235	0.84 (0.64–1.12)
GG	11 (2.0)	13 (2.3)	0.171	0.41 (0.12–1.46)	4 (1.6)	7 (2.9)	0.663	0.83 (0.37–1.89)
rs2839998								
GG	241 (46.1)	255 (46.2)		1.00	99 (41.4)	113 (47.9)		1.00
GA	222 (42.4)	243 (44.0)	0.255	1.26 (0.85–1.86)	108 (51.7)	101 (42.8)	0.786	0.97 (0.75–1.24)
AA	60 (11.5)	54 (9.8)	0.060	1.82 (0.98–3.41)	32 (13.4)	22 (9.3)	0.469	1.16 (0.77–1.75)
rs10376								
CC	448 (82.7)	447 (79.5)		1.00	210 (83.7)	193 (81.8)		1.00
AC	87 (16.1)	109 (19.4)	0.558	0.86 (0.52–1.43)	36 (14.3)	39 (16.5)	0.142	0.79 (0.58–1.08)
AA	7 (1.3)	6 (1.1)	0.942	0.95 (0.25–3.62)	5 (2.0)	4 (1.7)	0.812	1.14 (0.38–3.43)
rs2074733								
TT	166 (30.6)	156 (27.6)		1.00	55 (22.1)	65 (26.9)		1.00
TC	265 (48.8)	294 (51.9)	0.366	1.23 (0.79–1.92)	137 (55.0)	126 (52.1)	0.240	0.85 (0.64–1.12)
CC	112 (20.6)	116 (20.5)	0.284	1.34 (0.79–2.29)	57 (22.9)	51 (21.1)	0.550	0.90 (0.64–1.27)

a: Adjusted by age, sex and smoking

## Discussion

In the present study, we hypothesized that polymorphisms of *SF3A1* might contribute to the genetic susceptibility of CRC. Since the RNA splicing system is indispensable for functional diversity of protein and gene control in eukaryotic organisms, dysregulation of such machinery, including mutations of the core genes, can cause various diseases and cancers [37,38]. As a member of splicing complex, *SF3A1* has been implicated in various cancers. We thus proposed that polymorphisms might affect the *SF3A1* expression, then resulting in aberrant alternative splicing events in CRC.

The four candidate polymorphisms selected based on bioinformatics analysis and previous findings are located within non-coding regions of *SF3A1*. More than 1,200 GWAS studies have detected nearly 6,500 susceptibility loci[39] and it is noteworthy that 93% of which are located within non-coding regions[5]. Some of SNPs located within regulatory non-coding regions can affect gene expression and are major components in complex disease predisposition [40]. Among the four polymorphisms, rs10736, rs5753073 and rs2839998 are located in the 3’untranslated region (UTR), where microRNA binds and regulates the mRNA expression. These bindings can be affected by SNPs that reside in the microRNA target site, which can either abolish existing binding sites or create illegitimate binding sites, having a different effect on gene expression and representing another type of genetic variability that can influence the risk of certain human diseases[41]. Accumulative evidences have revealed that polymorphisms within micro-RNA-binding sites are associated with breast cancer[42], bladder cancer[43] and colorectal cancer[44]. In addition to microRNA target sites, the vast majority of CRC SNPs revealed by GWAS overlapped with at least one enhancer in colon crypt, some of which were significantly associated with low-frequency lost variant enhancer loci (VELs)[45] and linked to altered expression level of their target genes. Hence, the above researches suggested the putative roles of our non-coding SNPs in CRC carcinogenesis.

To investigate the roles of *SF3A1* polymorphisms in contributing to the susceptibility of CRC, we performed an association analysis of rs5753073, rs2839998, rs10376 and rs2074733 in 801 cases and 817 cancer-free controls in a Chinese population. However, all of these SNPs have failed to be associated with CRC risk. When stratified analyses were performed by the smoking and alcohol use status, we still obtained no statistically significant results. We proposed that the limited sample size might be insufficient for this association study, therefore more cases and controls are required in the future.

Some limitations also existed in our current study. First, as a hospital-based case-control study, selection bias might not avoid. Therefore, larger prospective studies are participated to confirm our results. Second, CRC is a heterogeneous disease in which environmental factors play an important role. Therefore, more other risk factors should be considered to further elucidate the etiology of CRC.

In summary, we firstly investigated the associations between polymorphisms of *SF3A1* and CRC risk. Our study demonstrated that rs5753073, rs2839998, rs10376 and rs2074733 did not confer to the risk of CRC based on the current hospital-based study. Certainly, larger population-based studies with comprehensive design are needed to further clarify the role of polymorphisms of *SF3A1* in the etiology of CRC.
